# Experimental toxocariasis in BALB/c mice: relationship between parasite inoculum and the IgG immune response

**DOI:** 10.1590/0074-02760160341

**Published:** 2017-03-27

**Authors:** Gabriela Rodrigues e Fonseca, Sergio Vieira dos Santos, Pedro Paulo Chieffi, Fabiana Martins de Paula, Ronaldo Cesar Borges Gryschek, Susana Angélica Zevallos Lescano

**Affiliations:** 1 Universidade de São PauloInstituto de Medicina Tropical de São PauloSão PauloSPBrasilInstituto de Medicina Tropical de São Paulo, São Paulo, SP, Brasil; 2 Irmandade da Santa Casa de Misericórdia de São PauloFaculdade de Ciências MédicasSanta Casa de São PauloSão PauloSPBrasilFaculdade de Ciências Médicas da Santa Casa de São Paulo, São Paulo, SP, Brasil; 3 Universidade de São PauloHospital das ClínicasFaculdade de MedicinaUniversidade de São PauloSão PauloSPBrasilHospital das Clínicas da Faculdade de Medicina da Universidade de São Paulo, Laboratório de Investigação Médica, São Paulo, SP, Brasil; 4 Universidade de São PauloUniversidade de São PauloFaculdade de MedicinaSão PauloSPBrasilUniversidade de São Paulo, Faculdade de Medicina, Programa de Pós-Graduação em Doenças Infecciosas e Parasitárias, São Paulo, SP, Brasil

**Keywords:** Toxocara canis, BALB/c, ELISA, larval recovery

## Abstract

BALB/c mice were inoculated with 5-500 *Toxocara canis* infective eggs, and bled at 15-120 days post infection (dpi) to evaluate the dynamics of IgG antibody response and larvae distribution. Positive results were observed in all occasions for every inoculum, and a direct proportional relationship between antibody detection and the parasitic load was observed. In samples collected at 60 dpi, detection of IgG was more intense, especially with the 50 and 500 egg doses; also, a correlation between antibody level and egg count was observed with these two inocula. At 120 dpi, a decrease in antibody titer was observed for all groups; and at the end of the experiment, larvae were recovered from carcass, liver and brain. In the liver, larvae were only found in mice inoculated with 500 *T. canis* eggs. In carcasses, these were recovered in all groups, and the group inoculated with 50 eggs showed the highest percentage of larvae in the brain.

*Toxocara canis* and *T. cati*, common ascarid nematode in dogs and cats, are the primary etiological agents of toxocariasis. In man, infection can provoke syndromes like visceral larva migrans (VLM), ocular lava migrans (OLM), covert toxocariasis (CT), and neurotoxocariasis (NT) (Chieffi et al. 2009, Fillaux & Magnaval 2013, Macpherson 2013, Overgaauw & van Knapen 2013). All of whose diagnoses mainly depend upon serological techniques (Moreira et al. 2014).

Even though most humans infected with *Toxocara* larvae present an asymptomatic course, some patients can present with significant symptomatic disease. Smith and Noordin (2006) reviewed the principal symptoms and signs present in the three clinical syndromes of human toxocariasis. These include VLM: fever, pallor, malaise, hepatomegaly, respiratory and nervous disturbance, irritability, weight loss, cutaneous rash, myocarditis, hypergammaglobulinemia, leukocytosis and eosinophilia, elevated anti-A and anti-B isohemagglutinins; OLM: visual loss, strabismus, endophthalmitis, chorioretinitis, uveitis, retinal granuloma and detachment; and CT: abdominal pain, weakness, headache, coughing, sleep and behavioral disturbances. Neurological syndromes (NS) observed during infection of the central nervous system (CNS) with *Toxocara* are generally nonspecific, and frequently, eosinophilia is a nonexistent sign (Chieffi et al. 2009).

*Toxocara* spp. infect two types of host: the definitive, especially dogs (*T. canis*) and cats (*T. cati*), and paratenic hosts (rodents, mammals and even invertebrates) (Macpherson 2013). A paratenic host is one in which parasite development does not occur, but may serve to bridge an ecological, or trophic, gap in a parasite’s life cycle (Bush et al. 2001). In paratenic hosts, *Toxocara* larvae penetrate the intestinal wall and, moving via the circulatory system, initiate the hepato-pulmonary phase, and advance to the myotropic-neurotropic phase, particularly accumulating in the brain and carcass (Abo-Shehada & Herbert 1984). They remain viable as encapsulated larvae for long time and, in this stage, they may continue their life cycle if the paratenic host is consumed as a prey by a definitive host (Strube et al. 2013).

Dubinský et al. (1995) considered mammals as important sources for *Toxocara* spp. maintenance under natural conditions and, furthermore, as an environmental indicator of contamination by these helminthes. Mice have been used as models for experimental toxocariasis studies because of their easy laboratory manipulation and the facility of tissue larval detection, as well as the ease of working with isogenic strains (Fan et al. 2015).

Studies in paratenic hosts, including mice, rats, rabbits, gerbils, and chickens, showed that *T. canis* larvae penetrate the small intestine walls when ingested and disseminate through the soft tissues of the body via systemic circulation. According to the literature, the most commonly affected organs are the liver, lung and eyes. The central nervous system, heart and skeletal muscles are affected less often (Camparoto et al. 2008).

Mice have frequently been used in studies investigating the migration and pathogenesis of this parasite. Strube et al. (2013) reported that haemorrhagic foci and parenchymatose congestion occurs in the lung and kidney. Also, larvae in the skeletal muscle and other organs, with exception of the CNS, were enclosed in granulomas of paratenic hosts. Examination of infected mice brains revealed demyelination, focal malacia and mixed cell infiltration, with areas of hemorrhage, congestion and neuronal necrosis. Similar neuropathological changes, predominantly in the myelinated tracts of the brain such as the corpus callosum, internal and external capsules, cerebellar peduncles and cerebellar medulla, have been described by other researchers, leading to the conclusion that larvae have an affinity for white matter of the brain. However, this rodent is not the best model for studying OLM (Akao et al. 2000); because, compared to mice, gerbils (*Meriones unguiculatus*) show 90% OLM incidence after oral infection resulting in pathomorphological signs including vitreous and retinal hemorrhage, vasculitis and exudative lesions.

The objective of the present study was to evaluate the humoral response using IgG antibodies in mice infected with different doses of embryonated *T. canis* eggs. The number of larvae recovered by tissue digestion will be compared at the end of the experiment.

Twenty five BALB/c Specific Pathogen-Free (SPF) male mice, 6-8 weeks old, acquired from Centro de Bioterismo of the Medicine Faculty of São Paulo University; were divided in four groups, three groups of six animals each were infected by intragastric intubation with 5, 50, or 500 embryonated *T. canis* eggs (L3), respectively, suspended in 200 μL of saline solution. Seven non-infected mice received 200 μL of saline solution and served as the negative control. Animals were kept in suitable light, temperature, and oxygen conditions, with water and food *ad libitum*.

Animals of all groups were anesthetised with xylazine and ketamine at concentrations of 10 mg/kg and 100 mg/kg, respectively, and were bled by retro orbital plexus at 15, 30, 60, 90, and 120 days post infection (dpi). Separated sera were frozen at -20ºC until processing.

Serum samples were examined by Enzyme-linked immunosorbent assay (ELISA) to detect anti-*Toxocara* IgG antibodies. The excreted *T. canis* antigen (TES-Ag) was prepared in this laboratory from cultures of infective larvae in Eagle medium following the standard technique of Bach-Rizzati (1984) and modified by Elefant et al. (2006). Different concentrations of ES antigen of *T*. *canis* were tested using positive (70 dpi from previous experiments) and negative control sera, and the protein concentration of 10 μg/mL (100 mL per well in plate) was determined for tests to be performed.

IgG ELISA: sera of mice were diluted in PBS-T-gel at 1:800; anti-mouse IgG conjugated with horseradish peroxidase (ɣ-chain specific, Sigma Immunochemicals) was used at a 1:4000 dilution. A mixture of H_2_O_2_ and O-Phenylenediamine (1,2-Diaminobenzene) [C_6_H_8_N_2_^.^2HCl] (Sigma Chemical Co.) diluted in citrate-phosphate buffer was used as the substrate.

Results were evaluated by spectrophotometric reading at a wavelength of 492 nm in the Titertek Multiskan apparatus MCC/3408 version 2.20 (LabSystems, Finland). To calculate the threshold of reactivity (“cut off”), the average optical density (OD) reading of sera from seven normal animals was determined, including two standard deviations (SD). As a positive control, serum samples from two mice with 70 days of infection were used and, as a negative control, serum samples from mice bled before infection were used. Statistical analysis of the ELISA test results was performed with the Kruskal-Wallis and multiple comparisons by Dunn’s test at p < 0.05.

At 170 dpi mice in Groups I, II, and III were euthanised and the carcass, liver and brain were removed. The artificial digestion of tissues was carried out in this laboratory using a modification of the procedure of Xi and Jin (1998). Organs were placed in a modified Baermann apparatus consisting of a beaker with a nylon strainer with 4 mm^2^ mesh; covered with a 0.5% hydrochloric acid (HCl) solution and incubated at 37ºC for 24 h. After this, the organs were discarded and pellets were centrifuged in conical tubes at 2000 rpm for two minutes, 2 mL of the sediment were collected, thoroughly mixed, and samples were observed under light microscope for larval counts.

This study received approval from the Ethics on Animal Use Committee of the Tropical Medicine Institute of São Paulo, Brazil (Protocol CPE-IMT/254).

There is a correlation between the level of IgG antibodies and the number of *T. canis* eggs inoculated in mice (Figure). The weakest antibody response was reached with an infective dose of five eggs with the maximal response occurring on 60 dpi.

The detection of IgG antibodies was at a minimum at the beginning of the infection period (15 dpi), with an increase beginning on the 30th dpi. A statistically significant difference was observed between groups I and III, and between group III and the control (p = 0.0003). Antibody levels peaked at 60 dpi, with a statistically significant difference between groups I and III, II and control, and III and control (p = 0.0003). After 90 dpi, the detection of IgG antibodies began to decline in group III, but there was an increase of antibody levels in groups I and II. There was a statistically significant difference between groups I and III, I and II, II and control, and III and control (p = 0.0006). At 120 dpi, a decrease in the antibody titers of all groups was observed (p = 0.0017). Table shows larval recovery at 170 dpi from diverse organs at the end of the experiment.

Larvae were recovered from the carcass in groups I, II and III with the following percentages: 6.67%, 8.33%, and 4.37% respectively. Larvae were only recovered from the liver in Group III. Group II presented the highest percentage of larvae found in the brain. Considering total recovery, there were 10% of larvae in group I, 14.33% in group II, and 7.14% in group III.

**TABLE I:** Media ± standard deviation (SD) and percentage of larvae of *Toxocara canis* recovered at 170 days post infection (dpi) in mice from groups I, II and III

	Carcass	Liver	Brain	Total
Group I	0.33 ± 0.51 (6.67%)	0 ± 0 (0.00%)	0.16 ± 0.40 (3.33%)	0.50 ± 0.83 (10.00%)
Group II	4.00 ± 1.72 (8.33%)	0 ± 0 (0.00%)	3.00 ± 2.00 (6.00%)	7.16 ± 2.13 (14.33%)
Group III	18.50 ± 15.14 (4.37%)	0.50 ± 0.54 (0.10%)	13.00 ± 3.66 (2.67%)	35.67 ± 17.75 (7.14%)

The ability of helminths to survive for long periods in the host depends on one of two factors: the host immune system must be depressed, or the invading agent has to possess adaptations that make it resistant to the attacks from the host immune response. In the case of *T. canis*, larvae that induce strong eosinophilic inflammation seem to be unaffected by this reaction, allowing the parasite to survive for several years in different host species (Lescano et al. 2012). Mice, tolerating massive *T. canis* infections for long periods, are considered appropriate models for better understanding of host-parasite relationships in human toxocariasis. In the present study, the correlation between the infection level and the humoral response was addressed, using different loads of *T. canis* eggs and detection of IgG antibodies under several conditions.

Murine models of helminthic infections have become very important to identify the protective mechanisms mediated by antibodies and the specific immune-effector cells that also contribute to protective immunity. Experiments with mice have provided data indicating that antibodies, particularly IgG and IgM, can act as potent mediators of protective immunity after infection with helminth parasites (Harris & Gause 2011). Pinelli et al. (2005) demonstrated that infection of BALB/c mice with *T. canis* results in chronic pulmonary inflammation and a dominant TH2 type immune response, regardless of inoculum size. Studies with plasma and bronchoalveolar fluid (BAL) of infected mice revealed increased inflammatory activity and an intense eosinophil migration that was associated with increased levels of cytokines like IL-6, IFN-γ, eotaxin and Regulated on Activation, Normal T Cell Expressed and Secreted (RANTES) in the infected rodents studied. Thus, establishing a strong correlation between tissue lesions caused by the larval migration and increased plasma levels of proinflammatory cytokines, as well as eosinophil chemotactic cytokines (Chieffi et al. 2009).

In this study, IgG antibodies were detected from the 15th dpi, and at the end of the experiment on 120 dpi, as shown in Figure. It is noteworthy that inoculum of only five *T. canis* eggs was sufficient to provoke a detectable immune response, although less intense than that evoked by the doses of 50 or 500 *T. canis* eggs.

**Figure:**
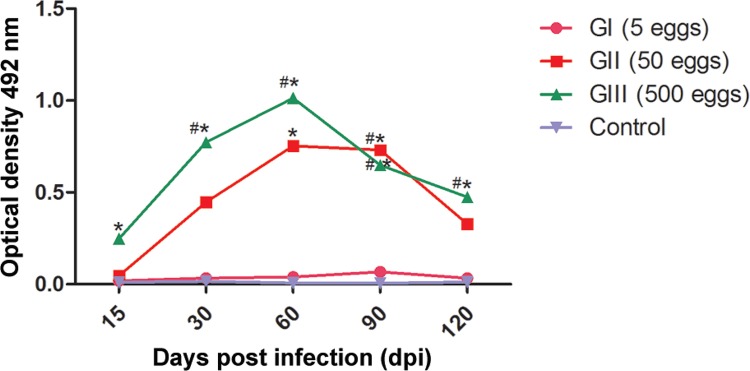
Enzyme-linked immunosorbent assay (ELISA) for detection of anti-*Toxocara* IgG. Each point represents the average value of optical density (OD) for six samples. *: significant difference between infected animals and control group; #: significant difference only among infected animals.

Studies indicate that recovery of *T. canis* larvae in mice depends on host strain, dose of inoculated eggs, and time post infection (Strube et al. 2013). The BALB/c strain is more susceptible to infection by *Toxocara* spp. (Bardón et al. 1994, Epe et al. 1994), with good larval recovery in the brain and greater survival rate when compared to other strains (Strube et al. 2013), justifying its use in the present assay. Infection of BALB/c mice with *T. canis* induces a dominant Th2 type immune response, with increased levels of total IgE and eosinophils; in tissues, microscopic lesions are represented by infiltrates of lymphocytes, neutrophils and eosinophils. This response however seems to be inefficient for the total elimination of larvae, which can survive in the tissues for long periods and re-migrate to other tissues (Cardillo et al. 2009), as confirmed in the larval recovery at the end of this experiment. By the time of larval recovery (170 dpi) their absence in the liver suggests that the parasites were already in the miotropic stage.

Some authors have reported the high affinity of *T. canis* larvae for the brain (Cuéllar et al. 1990, Guardis et al. 2002, Lescano et al. 2004, Janecek et al. 2014). This was also observed in this assay, albeit at less significant levels. This phenomenon also occurs by the localisation of the parasite in this organ, as the immune response may not encapsulate them into granulomas (Dunsmore et al. 1983, Guardis et al. 2002). Different researchers have reported recovery of larvae from the brain (Abo-Shehada & Herbert 1984, Guillén et al. 1990, Guardis et al. 2002, Ollero et al. 2008). However, in this study there was no direct proportional relationship to the parasite load in the brain and the number of inoculated *T. canis* larvae, as seen by the higher percentage found in the group infected with 50 ova.

Larvae were recovered in the carcasses of all groups; in absolute numbers, this recovery was directly proportional to the parasite load, but the percentage was highest in group II. By comparison, Bardón et al. (1994) observed larvae in the carcass after one year of infection that were proportionally less than the number found in the brain.

The host-parasite equilibrium could be a factor explaining the increased percentage of larvae recovered in group II. Group III was infected with a massive amount of embryonated eggs (500) and apparently, their immune response was more effective in eliminating the larvae. Even though the group II mice had a good immune response, they did not eliminate so many larvae, resulting in the highest percentage of observed recovery (Guardis et al. 2002).

Bardón et al. (1995) treated BALB/c mice infected with 1000 embryonated eggs with different doses of mebendazole, and demonstrated that the anthelmintic caused elimination and/or entrapment of some larvae and reduction in the levels of antibodies. However, larvae still released entrapped excretory-secretory products, which explains the persistence of the IgG response although at a lower level. The mice were not treated in this study, but it is possible that the elimination and or entrapping of larvae may have led to a decrease of antibody levels detected by the immunoassay at 120 dpi. In addition, antibody levels were shown to be dependent on egg dosage, as observed by other researchers (Smith & Noordin 2006, Janecek et al. 2014).

Cox and Holland (2001) suggested that the ingestion of low doses of eggs, with consequent lower load of *T. canis*, more realistically reflects the brain infection by this parasite in wild rodents and humans. The results of the present study suggest that mice of the Group I, infected with five eggs, possibly represents an infection similar to that which occurs in humans, especially in case of occult toxocariasis with detection of IgG antibodies and larval migration. This also raises the question of residual antibodies: are these the result of a past infection or do these patients still have a few larvae migrating through their tissues, releasing excretion-secretion products in small quantities, but still sufficient for detection by serological methods? Molecular biology studies might answer this question in the future.

*Ethical standards* - The authors assert that all procedures contributing to this work comply with the ethical standards of the relevant national and institutional guides on the care and use of laboratory animals, approved by the Ethics Committee on Animal Use in Tropical Medicine Institute of São Paulo Research No CPE-IMT/254.

## References

[B1] Abo-Shehada MN, Herbert IV. The migration of larval *Toxocara canis* in mice. II. Post-intestinal migration in primary infections. Vet Parasitol. 1984; 17(1): 75-83. 10.1016/0304-4017(84)90066-9 6543063

[B2] Akao N, Takayanagi TH, Suzuki R, Tsukidate S, Fujita K. Ocular larva migrans caused by *Toxocara cati* in Mongolian gerbils and comparison of ophthalmologic findings with those produced by *T. canis*. J Parasitol. 2000; 86(5): 1133-5. 10.1645/0022-3395(2000)086[1133:OLMCBT]2.0.CO;2 11128493

[B3] Bach-Rizzatti BC. Desenvolvimento do teste imunoenzimático ELISA para o diagnóstico da toxocaríase humana [PhD Thesis]. São Paulo: Universidade de São Paulo, Faculdade de Ciências Farmacêuticas da Universidade de São Paulo. 1984.

[B4] Bardón R, Cuéllar C, Del Aguila C, Guillen JL. Evaluation of mebendazole activity on experimental murine toxocariasis by immune complexes determination. Zentralbl Veterinarmed B. 1995; 42(4): 235-46. 10.1111/j.1439-0450.1995.tb00707.x 8546022

[B5] Bardón R, Cuéllar C, Guillén JL. Larval distribution of *Toxocara canis* in BALB/c mice at nine weeks and one year post-inoculation. J Helminthol. 1994; 68(4): 359-60. 10.1017/s0022149x00001644 7706687

[B6] Bush AO, Fernandez JC, Esch GW, Seed JR. Parasitism: the diversity and ecology of animal parasites. Cambridge: Cambridge University Press; 2001. 566 pp.

[B7] Camparoto ML, Fulan B, Colli CM, Paludo ML, Falavigna-Guilherme AL, Fernandez MA. Initial stage of development and migratory behavior of *Toxocara canis* larvae in BALB/c mouse experimental model. Genet Mol Res. 2008; 7(2): 444-50. 10.4238/vol7-2gmr443 18551411

[B8] Cardillo N, Rosa A, Ribicich M, López C, Sommerfelt I. Experimental infection with *Toxocara cati* in BALB/c mice, migratory behaviour and pathological changes. Zoonoses Public Health. 2009; 56(4): 198-205. 10.1111/j.1863-2378.2008.01182.x 18990197

[B9] Chieffi PP, Santos SV, Queiroz ML, Lescano SAZ. Human toxocariasis: contribution by Brazilian researchers. Rev Inst Med Trop São Paulo. 2009; 51(6): 301-8. 10.1590/s0036-46652009000600001 20209265

[B10] Cox DM, Holland CV. Influence of mouse strain infective dose and larval burden in the brain on activity in *Toxocara*-infected mice. J Helminthol. 2001; 75(1): 23-32. 10.1079/joh200027 11316469

[B11] Cuéllar C, Fenoy S, Guillén JL. Dinámica de la respuesta humoral en dos cepas murinas. I. Inoculación con huevos embrionados de *Toxocara canis*, *Toxascaris leonina* y *Ascaris suum*. Rev Iber Parasitol. 1990; 50(1-2): 137-50.

[B12] Dubinský P, Havasiová-Reiterová K, Petko B, Hovorka I, Tomasovicová O. Role of small mammals in the epidemiology of toxocariasis. Parasitology. 1995; 110(Pt 2): 187-93. 10.1017/s0031182000063952 7885737

[B13] Dunsmore JD, Thompson RC, Bates IA. The accumulation of *Toxocara canis* larvae in the brains of mice. Int J Parasitol. 1983; 13(5): 517-21. 10.1016/s0020-7519(83)80017-4 6642866

[B14] Elefant GR, Shimizu SH, Sanchez MCA, Jacob CMA, Ferreira AW. A serological follow-up of toxocariasis patients after chemotherapy based on the detection of IgG, IgA and IgE antibodies by enzyme-linked immunosorbent assay. J Clin Lab Anal. 2006; 20(4): 164-72. 10.1002/jcla.20126 PMC6807646 16874812

[B15] Epe C, Sabel T, Schnieder T, Stoye M. The behavior and pathogenicity of *Toxocara canis* larvae in mice of different strains. Parasitol Res. 1994; 80(8): 691-5. 10.1007/BF00932955 7886040

[B16] Fan CK, Holland CV, Loxton K, Barghout U. Cerebral toxocariasis: silent progression to neurodegenerative disorders? Clin Microbiol Rev. 2015; 28(3): 663-86. 10.1128/CMR.00106-14 PMC4462679 26062575

[B17] Fillaux J, Magnaval JF. Laboratory diagnosis of human toxocariasis. Vet Parasitol. 2013; 193(4): 327-36. 10.1016/j.vetpar.2012.12.028 23318165

[B18] Guardis MV, Radman NE, Burgos L, Fonrouge RD, Archelli SM. *Toxocara canis*: migración larval y eosinofilia en el hospedador paraténico. Parasitol Latinoam. 2002; 57(1-2): 46-9.

[B19] Guillén J, Bardón R, Domínguez P, Cuéllar C. Migración larvaria de *Toxocara canis* y respuesta eosinofilica en las cepas murinas BALB/c y C57BL/10. Rev Iber Parasitol. 1990; 50(3-4): 289-99.

[B20] Harris N, Gause WC. To B or not to B: B cells and Th2-type immune response to helminths. Trends Immunol. 2011; 32(2): 80-8. 10.1016/j.it.2010.11.005 PMC3076625 21159556

[B21] Janecek E, Beineke A, Schnieder T, Strube C. Neurotoxocarosis: marked preference of *Toxocara canis* for the cerebrum and *T. cati* for the cerebellum in the paratenic model host mouse. Parasit Vectors. 2014; 7: 194. 10.1186/1756-3305-7-194 PMC4017833 24754900

[B22] Lescano SAZ, Nakhle MC, Ribeiro MCS, Chieffi PP. IgG antibody responses in mice coinfected with *Toxocara canis* and other helminths or protozoan parasites. Rev Inst Med Trop São Paulo. 2012; 54(3): 145-52. 10.1590/s0036-46652012000300006 22634886

[B23] Lescano SZ, Queiroz ML, Chieffi PP. Larval recovery of *Toxocara canis* in organs and tissues of experimentally infected *Rattus norvegicus*. Mem Inst Oswaldo Cruz. 2004; 99(6): 627-8. 10.1590/s0074-02762004000600016 15558175

[B24] Macpherson CN. The epidemiology and public health importance of toxocariasis: a zoonosis of global importance. Int J Parasitol. 2013; 43(12-13): 999-1008. 10.1016/j.ijpara.2013.07.004 23954435

[B25] Moreira GM, Telmo PL, Mendonça M, Moreira AN, McBride AJ, Scaini CJ, et al. Human toxocariasis: current advances in diagnostics, treatment and interventions. Trends Parasitol. 2014; 30(9): 456-64. 10.1016/j.pt.2014.07.003 25089038

[B26] Ollero MD, Fenoy S, Cuéllar C, Guillén JL, Del Aguila C. Experimental toxocariosis in BALB/c mice: effect of the inoculation dose on brain and eye involvement. Acta Trop. 2008; 105(2): 124-30. 10.1016/j.actatropica.2007.11.001 18093569

[B27] Overgaauw PA, van Knapen F. Veterinary and public health aspects of *Toxocara* spp. Vet Parasitol. 2013; 193(4): 398-403. 10.1016/j.vetpar.2012.12.035 23305972

[B28] Pinelli E, Withagen C, Fonville M, Verlaan A, Dormans J, van Loveren H, et al. Persistent airway hyper-responsiveness and inflammation in *Toxocara canis*- infected BALB/c mice. Clin Exp Allergy. 2005; 35(6): 826-32. 10.1111/j.1365-2222.2005.02250.x 15969676

[B29] Smith HV, Noordin R. Diagnostic limitations and future trends in the serodiagnosis of human toxocariasis. In: Smith HV, Holland CV, editors. *Toxocara, the enigmatic parasite*. Wallingford: CABI Publishing; 2006. p. 89-112.

[B30] Strube C, Heuer L, Janecek E. *Toxocara* spp. infections in paratenic hosts. Vet Parasitol. 2013; 193(4): 375-89. 10.1016/j.vetpar.2012.12.033 23312872

[B31] Xi WG, Jin LZ. A novel method for the recovery of *Toxocara canis* in mice. J Helminthol. 1998; 72(2): 183-4. 10.1017/s0022149x00016382 9687601

